# Parental occupational exposure and congenital heart diseases in a Hungarian case–control study

**DOI:** 10.1007/s00420-020-01589-4

**Published:** 2020-11-10

**Authors:** Vince Fazekas-Pongor, Mónika Fekete, Melinda Csáky-Szunyogh, Károly Cseh, Melinda Pénzes

**Affiliations:** 1 Department of Public Health, Faculty of Medicine, Semmelweis University Budapest, Üllői út 26, Budapest, 1085 Hungary; 2 Hungarian Congenital Abnormalities Registry, National Public Health Center, Albert Flórián út 2-6, Budapest, 1097 Hungary

**Keywords:** Endocrine disruptor, Congenital heart disease, Retrospective exposure assessment, Occupational exposure, Maternal exposure, Paternal exposure

## Abstract

**Purpose:**

Our study aimed to explore the effect of parental occupational exposure to endocrine disrupting chemicals (EDCs) on the development of congenital heart diseases (CHDs) in the offspring, and to compare job-exposure matrix (JEM)-assessed and self-reported occupational exposures with each other.

**Methods:**

Live-born infants born in 2007–2008 were selected from the population-based Hungarian Case–Control Surveillance of Congenital Abnormalities Study. 577 cases with any CHDs were compared to 1731 matched controls. Parental periconceptional occupational exposure to EDCs was assessed by a JEM and by questionnaire-based self-reporting of parents. Multivariate conditional logistic regression analyses were conducted to explore associations between parental occupational exposure to EDCs and the entire spectrum of CHDs and by CHD subtypes in the offspring. Kappa statistics were also performed to determine the consistency among JEM-assessed and self-reported occupational exposure of parents.

**Results:**

JEM-assessed paternal exposure to polychlorinated organic substances, phthalates, biphenolic compounds, and solvents were significantly associated with the entire spectrum of CHDs. Ventricular septal defects were significantly associated with paternal self-reported exposure to pesticides, while atrial septal defects were significantly associated to paternal JEM-assessed phthalate exposure. Paternal solvent exposure was significantly associated with atrial septal defects and right ventricle outflow tract obstructions. JEM-assessed and self-reported exposures to pesticides, heavy metals, and solvents exhibited poor agreement for mothers and slight agreement for fathers.

**Conclusion:**

Even though parental occupational exposure to EDCs seems to have a minor impact on the occurrence of CHDs, the results of biological and environmental monitoring should be taken into consideration as well.

## Introduction

Congenital anomalies are defined as either structural or functional anomalies occurring during the intrauterine life (WHO 2016). Congenital heart diseases (CHDs) are among the most common congenital birth defects (WHO 2016), and affect approximately 1% of live births (Nicoll 2018). In Europe, the live birth prevalence of CHDs is 7.2 per 1000 births (Dolk et al. 2011). In the annual report of the European Surveillance of Congenital Anomalies, an increase in birth prevalence has been observed for the entire spectrum of CHDs with a marked increase for abnormalities such as single ventricle or Tetralogy of Fallot (EUROCAT 2014). According to a study on the trends of congenital anomalies from 1980 to 2012, Hungary is a frequent outlier among other European countries with a much higher rate of CHDs (14.5 cases per 1000 live births) (Morris et al. 2018; EUROCAT 2020). In addition, certain CHD subtypes such as patent ductus arteriosus have also been increasing in contrast to trends observed elsewhere (Morris et al. 2018). The exact causes of these differences are not fully understood yet.

Approximately, 20% of CHDs are linked to known causes (Fung et al. 2013). The etiology of the rest is unknown and may be a result of complex interactions between environmental and genetic factors (Snijder et al. 2012). Pregnancy-related factors, like parity [adjusted odds ratio (aOR) = 1.18; 95% confidence interval (CI) 1.03–1.34], past induced abortion (aOR 1.58; 95% CI 1.12–2.22) or the use of assisted reproduction techniques for conception (aOR 1.45; 95% CI 1.20–1.76) may increase the risk of CHDs (Feng et al. 2015; Giorgione et al. 2018). Socio-demographic factors of parents, such as low education (aOR 1.47; 95% CI 1.28–1.69) or high maternal age (aOR 1.94; 95% CI 1.12–3.34), and lifestyle habits, such as smoking (aOR 1.11; 95% CI 1.02–1.21) or alcohol misuse (aOR 2.14; 95% CI 1.64–2.8), were also linked to the development of certain CHDs (Ou et al. 2016; Nicoll 2018). Other factors that may influence CHD outcomes include higher maternal body mass index (aOR 1.32; 95% CI 1.21–1.43), maternal diabetes (aOR 4.12; 95% CI 3.69–4.60), maternal hypertension (Relative Risk = 2.00; 95% CI 1.5–2.7), maternal antibiotic use during pregnancy (aOR 3.96; 95% CI 1.78–8.79), maternal anticonvulsant medication use during pregnancy (aOR 2.49; 95% CI 1.47–4.21), and periconceptional folate intake (aOR 0.42; 95% CI 0.21–0.86) (Liu et al. 2013; Ban et al. 2015; Ramakrishnan et al. 2015; Ou et al. 2016; Mao et al. 2017; Zheng et al. 2018).

Studies suggest that the occupation of parents may also increase the occurrence of CHDs (Peng et al. 2019). Offspring of professionals exposed to endocrine disrupting chemicals are especially vulnerable to develop CHDs. Endocrine disrupting chemicals are used in numerous occupations, for instance among painters, farmers, metalworkers, or woodworkers (Snijder et al. 2012). Exposure to certain endocrine disrupting chemicals like phthalates (aOR 2.08; 95% CI 1.27–3.40), alkylphenolic compounds (aOR 3.85; 95% CI 1.17–12.67), pesticides (aOR 1.7; 95% CI 1.34–2.14), solvents (aOR 1.6; 95% CI 1.0–2.6), and polychlorinated organic compounds (aOR 4.22; 95% CI 1.23–14.42) have all been linked to CHDs (Wilson et al. 1998; Snijder et al. 2012; Nicoll 2018). Phthalates frequently occur in plastics, cosmetics, paints, and electronic devices, whereas alkylphenolic compounds are primarily found in surfactants, emulsifiers, detergents, and pesticides (IARC 2012; Bergman et al. 2013; Ye et al. 2014; Pecht et al. 2018). Biphenolic compounds are often used in polycarbonates and epoxy resins (Russo et al. 2019), while polychlorinated organic compounds are utilized regularly in electrical equipment, pigments, and plasticizers (EPA 2019). Finally, pesticide exposure is especially common among farmers, who come into contact with a wide variety of pesticides in far greater concentrations than consumers (Damalas and Koutroubas 2016).

The mechanism how these occupational factors may lead to the development of CHDs is not fully understood yet. Many endocrine disrupting chemicals have an estrogenic effect and may lead to impaired semen quality (Jeng 2014), disrupted oocyte maturation (Gregoraszczuk and Ptak 2013), or fetal epigenomic changes (Bommarito et al. 2017). Polychlorinated compounds can induce oxidative stress (Zhu et al. 2009), which has also been associated with CHDs (Wu et al. 2016). Pesticides have a more complex effect, they not only disrupt the endocrine system but also interfere with the proper function of the immune system and neurodevelopment, moreover, they are carcinogenic as well (Kalliora et al. 2018). Even though biphenolic compounds have not been widely associated to CHDs in humans, animal studies indicate that biphenolic compounds may also disturb the process of cardiogenesis and lead to transgenerational inheritance of CHDs (Lombó et al. 2015).

Since biological and environmental monitoring are often expensive and the available data is either limited, incomplete, or not gathered for scientific purposes, job-exposure matrices (JEMs) and self-reported occupational exposure questionnaires are frequently used as an alternate (Teschke et al. 2002; Snijder et al. 2012). JEMs estimate occupational exposures by assigning probability of exposure to job titles (Fischer et al. 2017) and have been used in the past to identify possible occupational exposure to endocrine disrupting chemicals behind congenital malformations such as CHDs (Snijder et al. 2012). In addition, JEMs may be useful in reducing recall bias associated with other survey instruments (Liew et al. 2014). Based on studies conducted in the past, both JEMs and self-reported occupational exposure questionnaires can be used as surrogates for the assessment of occupational exposure even though the agreement between aforementioned methods may vary (Teschke et al. 2002; Adegoke et al. 2004).

Studies indicate that occupational exposure to EDCs may increase the occurrence of CHDs in the offspring. Thus, the aims of our study were (1) to identify possible parental occupational exposures to endocrine disrupting chemicals predisposing the fetus to the development of CHDs, and (2) to compare JEM-assessed and self-reported occupational exposures with each other.

## Methods

### Study design and population

In Hungary, reporting of cases with congenital anomalies is mandatory for physicians, and most are reported by obstetricians and pediatricians to the Hungarian Congenital Abnormality Registry (HCAR). Reporting rates are high as physicians are legally obliged to report congenital malformations within 1 month of diagnosis. If reporting willingness does not reach the expected level in certain areas, an audit is performed along with the request of follow-up data supplementation.

The Hungarian Case–Control Surveillance of Congenital Abnormalities Study (HCCSCAS) was started in 1980 to identify the causes of these disorders and to investigate the possible causality of environmental and parental conditions during pregnancy. All cases included in the HCCSCAS are selected from the HCAR. Only live births reported to the HCAR within the first 3 months of diagnosis are recruited. Any cases reported after that are excluded from the study to minimize the effect of recall bias. Syndromes and any malformations with genetical background are also excluded from the HCCSCAS. Twin births, on the other hand, are not excluded and are always indicated in the database. Controls are defined as newborns without any congenital malformation and are selected within 3 months following the reporting of cases. Controls are matched to cases according to their gender, date of birth, and area of parents’ residence. If controls are twins, only one of the twins is customarily recruited into the database. In Hungary, families meet their correspondent district nurse at a regular, almost monthly basis during pregnancy and within the first year of birth. As a part of the HCCSCAS, case and control families are interviewed by the district nurse, who are already aware of potential risky behavior of parents, minimizing the effect of recall bias concerning risk factors that are seen negatively by society, for instance smoking during pregnancy. Families receive oral and written information about the study and its goal, then sign an informed consent before data collection. Consent forms were signed by 98% of the participants. A single interview is conducted with families using printed questionnaires. Pregnancy data and medications are cross-validated with the prenatal logbook.

In our present study, we decided to focus on data obtained between years 2007 and 2008 because periconceptional occupational exposure was assessed in depths only during this time period. A total of 577 cases of CHDs and 1731 controls were enrolled in our present analysis. Case and control newborns were matched at a ratio of 1:3. CHDs were classified by the tenth revision of the International Classification of Diseases. We examined the entire spectrum of CHDs (Q20.0–Q26.4) and the following subtypes separately: atrial septum defects (Q21.1), ventricular septum defects (Q21.0), right ventricle outflow tract obstructions, left ventricle outflow tract obstructions, and patent ductus arteriosus (Q25.0). Right ventricle outflow tract obstructions are an umbrella term for anomalies such as pulmonary atresia (Q25.5), pulmonary stenosis (Q25.6), pulmonary valve stenosis (Q22.1), pulmonary valve atresia (Q22.0) and Tetralogy of Fallot (Q21.3) (Thorne and Clift 2011a), whereas left ventricle outflow tract obstructions are comprised of malformations including aortic valve stenosis (Q23.0), stenosis of the aorta (Q25.3), atresia of aorta (Q25.2), and coarctation of the aorta (Q25.1) (Thorne and Clift 2011b).

### Measurement

In the present study, the following pregnancy-related variables were assessed: birth weight and gestational age of newborns, parity (primipara/multipara), past induced abortion (yes/no), the use of assisted reproduction techniques for the present conception (yes/no), occurrence of twin birth (yes/no), and family history of congenital malformations (yes/no). The questionnaire also explored parental socio-demographic, lifestyle, and health-related factors, including maternal and paternal age, maternal and paternal education (less than primary, primary—primary school, secondary—high school or vocation school with graduation certificate, or tertiary education—college or university), maternal body mass index (< 24.99, 25.00–29.99, > 30.00 kg/m^2^), gestational or pregestational maternal diabetes (yes/no), gestational or pregestational maternal hypertension (yes/no), maternal and paternal smoking during pregnancy (yes/no), maternal and paternal alcohol consumption during pregnancy (yes/no), gestational antibiotic use (yes/no), gestational anticonvulsant medication use (yes/no), and periconceptional maternal folic acid intake (yes/no). Settlement size of family’s residence was coded according to the Hungarian governmental settlement hierarchy into following groups: < 5000, 5000–20,000, 20,000–100,000, 100,000–1,000,000, and > 1,000,000 inhabitants.

Periconceptional occupational exposure was estimated by evaluating parental job titles and by self-reported risk assessment of parents. Parents were asked to state their job during the periconceptional time period and give a brief description of their tasks. Periconceptional period was defined as 1 month prior and 2 months after the calculated time of conception. Job titles were coded by the Hungarian Occupational Exposure System which is based on the International Standard Classification of Occupations (ISCO-08). The Hungarian Occupational Exposure System contains 485 individual job titles entailing the most important occupations in Hungary. Job titles were then categorized by Van Tongeren’s et al.’s (Van Tongeren et al. 2002) JEM as either no exposure (0), possible exposure (1), or probable exposure (2) to pesticides, polychlorinated organic compounds, alkylphenolic compounds, phthalates, biphenolic compounds, and heavy metals. Van Tongeren’s et al.’s (2002) JEM is based on independent expert assessment of job titles as either probable, possible, or unlikely exposure to endocrine disrupting chemicals. Following Snijder et al.’s (2002) recommendation, we decided to collapse probable and possible exposures into a single category for our subtype analysis in order to decrease the number of concurrent analyses (Snijder et al. 2012). Since van Tongeren’s et al.’s (2002) JEM does not evaluate exposure to solvents, we used the Nordic Occupational Cancer Study’s JEM to categorize occupations in regards of possible or probable exposure to the following solvents: aliphatic and alicyclic hydrocarbon solvents, aromatic hydrocarbon solvents, benzene, toluene, chlorinated hydrocarbon solvents, perchloroethylene, 1,1,1 trichloroethane, and trichloroethylene (Hadkhale et al. 2017). The evaluation of job titles included in the Hungarian Occupational Exposure System was conducted independently by two of the authors (F-P V, Cs K). Interrater reliability was assessed by calculating kappa (*κ*) values, which were interpreted according to the following ranges: < 0, poor agreement; 0.0–0.20, slight agreement; 0.21–0.40, fair agreement; 0.41–0.60, moderate agreement; 0.61–0.80, substantial agreement; 0.81–1.00, almost perfect agreement (Landis and Koch 1977). The agreement in case of pesticides (*κ* = 0.91), polychlorinated organic compounds (*κ* = 0.75), phthalates (*κ* = 0.89), alkylphenolic compounds (*κ* = 0.94), biphenolic compounds (*κ* = 0.91), heavy metals (*κ* = 0.85), and solvents (*κ* = 0.81) ranged from moderate to almost perfect agreement. Disagreements were discussed prior to the creation of the final matrix. Exposure assessment of parents included in the study was blinded to both the outcome and participants.

Finally, as part of the occupational exposure assessment, parents were also asked to report their self-perceived periconceptional occupational exposure in regards of pesticides, solvents, plastics, heavy metals, and other substances (Question: “During the periconceptional period, were you exposed to one or several of these compounds at your workplace?”). Other substances were defined as any other chemical exposure not listed above. Self-reported occupational exposures were named differently than in the JEM because reliability of self-reported questionnaires tends to increase if risk factors appear in ways that are easier to recognize for the workers (Teschke et al. 2002). The results were coded into a dichotomous variable (yes/no).

### Statistical analysis

Descriptive characteristics of cases and controls were compared by using Mann–Whitney *U* test for continuous and Chi-squared test for categorical variables. Series of univariate conditional logistic regression analyses were also performed to test associations between socio-demographic, pregnancy-related, and parental lifestyle factors and CHDs. Separate multivariate conditional logistic regression analyses were then conducted to explore individual associations between maternal and paternal occupational exposure to endocrine disrupting chemicals and the entire spectrum of CHDs in the offspring and by CHD subtypes mentioned above. The selection of potential confounders was based on the significant results of the univariate conditional logistic regression analyses. For mothers, the following confounders were selected: maternal age, maternal education, parity, past induced abortion, family history of congenital anomalies, maternal body mass index, gestational or pregestational diabetes, gestational or pregestational hypertension, maternal smoking, and periconceptional maternal folic acid intake. For fathers, the following confounders were selected: paternal age, paternal education, family history of congenital anomalies, and paternal smoking. Gestational age and birthweight were not included in our regression models, as they are consequences of CHDs and not risk factors of their development. Adjusted odds ratios (aOR) were calculated in the multivariate conditional logistic regression analyses by correcting for the aforementioned confounders. If associations were significant in the multivariate analyses, the job titles involved were identified.

Finally, Kappa statistics were performed to determine consistency among JEM-assessed and self-reported occupational exposure of both mothers and fathers. Direct comparison could be made only in cases of pesticides, heavy metals, and solvents because of the different grouping methods used for the JEM and self-reported occupational exposure assessment. Significance level was set at *p* < 0.05. All analyses were performed using IBM SPSS 24.0.

## Results

General characteristics of cases and controls and the results of the univariate conditional logistic regression analyses are displayed in Table 1. Case newborns exhibited significantly lower birth weight and gestational age compared to controls and significantly higher occurrence of any congenital anomaly in their family history. Case mothers significantly differed in their education, parity, occurrence of past abortion, occurrence of gestational or pregestational diabetes and hypertension, periconceptional folate intake, and smoking habits compared to control mothers. Case fathers were significantly different in their education and smoking habits compared to control fathers. In the univariate conditional regression analyses, birth weight and gestational age of newborns, family history of any congenital anomaly, maternal age, maternal education, parity, past abortion, periconceptional folic acid intake, maternal body mass index, gestational or pregestational diabetes, gestational or pregestational hypertension, maternal smoking, paternal education, and paternal smoking showed significant association with the entire spectrum of CHDs in the offspring (Table 1).

**Table 1:** General characteristics of families of case and control newborns and associations between these characteristics and the entire spectrum of CHDs in the offspring in univariate logistic regression analyses

	Cases (*n* = 577)	Controls (*n* = 1731)	OR^b^ (CI 95%)
	*n* (%)	*n* (%)	
Characteristics of newborns			
Gender			
Male	284 (49.2)	852 (49.2)	N/A^c^
Female	293 (50.8)	879 (50.8)	
Mean birth weight in grams (SD)	3200 (708)	3339 (548)	0.99 (0.98–0.99) ^b^
Birth weight > 2500g^a^	488 (84.6)	1646 (95.1)	Ref.
LBW^a^	89 (15.4)	85 (4.9)	3.40 (2.49–4.65)^b^
Mean gestational age in weeks (SD)^a^	38.5 (2.2)	38.96 (1.7)	0.88 (0.84–0.92)^b^
Twins (yes)	15 (2.6)	30 (1.7)	1.51 (0.81–2.82)
CHD			
Isolated	398 (69.0)	–	–
Multiple	179 (31.0)	–	–
Family history of CAs (yes)^a^	28 (4.9)	38 (2.2)	2.33 (1.40–3.88)^b^
Characteristics of mother			
Age (years) (SD)	29.3 (5.6)	28.73 (3.8)	1.03 (1.01–1.06)^b^
Education level^a^			
Less than primary	24 (4.2)	25 (1.4)	3.86 (2.13–7.00)^b^
Primary	99 (17.2)	197 (11.4)	2.08 (1.50–2.88)^b^
Secondary	308 (53.4)	968 (55.9)	1.22 (0.97–1.54)
Tertiary	146 (25.3)	541 (31.3)	Ref.
Parity^a^			
Primipara	436 (75.6)	1447 (83.6)	Ref.
Multipara	141 (24.4)	284 (16.4)	1.70 (1.34–2.15)^b^
Pregnancy			
Twins (yes/no)	15 (2.6)	30 (1.7)	1.51 (0.81–2.82)
Previous abortion (yes)^a^	131 (22.7)	309 (17.9)	1.35 (1.07–1.70)^b^
Assisted reproduction (yes)	40 (6.9)	105 (6.1)	1.16 (0.79–1.71)
Folic acid (yes)^a^	315 (54.6)	1272 (73.5)	0.40 (0.32–0.49)^b^
BMI (kg/m^2^)^a^			
< 24.99	384 (66.6)	1212 (70.0)	Ref.
25–29.99	114 (19.8)	339 (19.6)	1.07 (0.83–1.36)
> 30	79 (13.7)	180 (10.4)	1.39 (1.04–1.86)^b^
Pregestational/gestational diabetes (yes)^a^	37 (6.4)	63 (3.6)	1.79 (1.19–2.71)^b^
Pregestational/gestational hypertension (yes)^a^	30 (5.2)	58 (3.4)	1.59 (1.01–2.50)^b^
Smoking (yes)^a^	119 (20.6)	210 (12.1)	1.88 (1.46–2.40)^b^
Alcohol consumption(yes)	37 (6.4)	100 (5.8)	1.12 (0.76–1.64)
Antibiotic use (yes)	52 (9.0)	198 (11.4)	1.33 (0.95–1.86)
Anticonvulsant medication use (yes)	2 (0.3)	6 (0.3)	1.00 (0.20–4.96)
Characteristics of father			
Age (years) (SD)	32.1 (6.5)	31.97 (5.3)	1.00 (0.99–1.02)
Education level^a^			
Less than primary	28 (4.9)	33 (1.9)	3.40 (1.90–6.07)^b^
Primary	107 (18.5)	198 (11.4)	2.05 (1.46–2.87)^b^
Secondary	331 (57.4)	1116 (64.5)	1.07 (0.84–1.38)
Tertiary	111 (19.2)	384 (22.2)	Ref.
Smoking (yes)^a^	293 (50.8)	698 (40.3)	1.56 (1.27–1.90)^b^
Alcohol consumption (yes)	382 (66.2)	1150 (66.4)	0.99 (0.81–1.21)
Settlement size of family			
< 5000	168 (29.1)	504 (29.1)	N/A^c^
5000–20,000	24 (4.2)	72 (4.2)	
20,000–100,000	193 (33.4)	579 (33.4)	
100,000–1,000,000	161 (27.9)	483 (27.9)	
> 1,000,000	31 (5.4)	93 (5.4)	

*BMI* body mass index, *CA* congenital anomaly, *CHD* congenital heart disease, *CI* confidence interval, *LBW* low birth weight, *OR* odds ratio, *Ref*. reference, *SD* standard deviation

^a^Mann–Whitney *U* test or Chi squared test, *p* **< **0.050

^b^Univariate logistic regression model, *p* **< **0.050

^c^N/A: Not applicable due to matching criteria between cases and controls

Based on the results of the JEM, 33.6% and 31.4% of case and control mothers, while 21.4% and 18.6% of case and control fathers, respectively, worked in areas where exposure to endocrine disrupting chemicals is either possible or probable. As for self-reported occupational exposure, 27.9% and 29.1% of case and control mothers, and 41.1% and 41.5% of case and control fathers, respectively, reported possible exposure to certain occupational risk factors. Detailed information on maternal and paternal possible and probable occupational exposures to individual endocrine disrupting chemicals are displayed in Table 2, while detailed information on parental self-reported exposure prevalence data are summarized in Table 3.

**Table 2:** Prevalence of JEM-assessed parental occupational exposure and associations between periconceptional parental JEM-assessed occupational exposure to endocrine disrupting chemicals and the entire spectrum of CHDs in the offspring

	Possible exposure				Probable exposure			
	Exposure prevalence		CHDs		Exposure prevalence		CHDs	
	Cases, *n* (%)	Controls, *n* (%)	OR (95% CI)	aOR (95% CI)	Cases, *n* (%)	Controls, *n* (%)	OR (95% CI)	aOR (95% CI)
JEM-assessed maternal occupational exposure								
Pesticides	–	7 (0.4)	0.03 (0.00–46.0)	–	4 (0.7)	14 (0.8)	0.86 (0.28–2.60)	1.06 (0.33–3.40)
POCs	1 (0.2)	2 (0.1)	1.50 (0.14–16.5)	1.17 (0.08–16.2)	2 (0.3)	2 (0.1)	3.00 (0.42–21.3)	3.02 (0.38–24.0)
Phthalates	13 (2.3)	45 (2.6)	0.86 (0.46–1.62)	0.78 (0.40–1.53)	22 (3.8)	42 (2.4)	1.61 (0.95–2.73)	1.68 (0.96–2.95)
APCs	6 (1.0)	23 (1.3)	0.78 (0.32–1.92)	0.93 (0.36–2.39)	17 (2.)	50 (2.9)	1.02 (0.58–1.79)	1.04 (0.57–1.88)
BPCs	–	–	–	–	2 (0.3)	5 (0.3)	1.20 (0.23–6.19)	1.08 (0.18–6.36)
Heavy metals	1 (0.2)	2 (0.1)	1.50 (0.14–16.5)	1.35 (0.09–19.6)	9 (1.6)	22 (1.3)	1.16 (0.60–2.25)	1.13 (0.50–2.57)
Solvents	10 (0.6)	3 (0.5)	0.92 (0.30–2.87)	1.09 (0.29–4.08)	145 (25.1)	394 (22.8)	1.10 (0.91–1.33)	1.19 (0.95–1.51)
JEM-assessed paternal occupational exposure								
Pesticides	3 (0.5)	25 (1.4)	0.36 (0.11–0.19)	0.30 (0.08–1.00)	21 (3.6)	39 (2.3)	1.65 (0.96–2.83)	1.58 (0.90–2.77)
POCs	4 (0.7)	11 (0.6)	1.09 (0.35–3.43)	1.16 (0.37–3.69)	12 (2.1)	18 (1.0)	2.04 (0.97–4.28)	2.25 (1.05–4.82)^a^
Phthalates	1 (0.2)	2 (0.1)	1.50 (0.14–16.5)	1.85 (0.17–20.6)	34 (5.9)	64 (3.7)	1.61 (1.06–2.46)^a^	1.72 (1.11–2.65)^a^
APCs	25 (4.3)	66 (3.8)	1.14 (0.71–1.83)	1.01 (0.67–1.79)	3 (0.5)	5 (0.3)	1.80 (0.43–7.53)	1.72 (0.38–7.74)
BPCs	–	–	–	–	3 (0.5)	1 (0.1)	9.00 (0.94–86.5)	11.5 (1.17–113)^a^
Heavy metals	2 (0.3)	19 (1.1)	0.32 (0.07–1.36)	0.32 (0.07–1.39)	43 (7.5)	128 (7.4)	1.01 (0.70–1.46)	1.12 (0.77–1.64)
Solvents	27 (4.79	69 (4.0)	1.13 (0.77–1.67)	1.25 (0.78–1.99)	23 (4.0)	35 (2.0)	1.61 (1.06–2.44)^a^	2.16 (1.24–3.76)

Maternal occupational exposure was adjusted for family history of congenital anomalies, maternal age, maternal education, parity, past abortion, maternal body mass index, gestational or pregestational diabetes, gestational or pregestational hypertension, maternal smoking, and periconceptional folic acid intake

Paternal occupational exposure was adjusted for family history of congenital anomalies, paternal age, paternal education, and paternal smoking

*APCs* alkylphenolic compounds, *BPCs* biphenolic compounds, *CHDs* congenital heart diseases, *CI* confidence interval, *JEM* job-exposure matrix, *OR* odds ratio, *aOR* adjusted odds ratio, *POCs* polychlorinated organic compounds

^a^*p* **< **0.050

**Table 3:** Prevalence of self-reported parental occupational exposure and associations between periconceptional parental self-reported occupational exposure to endocrine disrupting chemicals and the entire spectrum of CHDs in the offspring

	Exposure Prevalence		CHDs	
	Cases, *n* (%)	Controls, *n* (%)	OR (95% CI)	aOR (95% CI)
Self-reported maternal occupational exposure				
Pesticides	4 (0.7)	19 (1.1)	0.63 (0.22–1.86)	0.54 (0.18–1.63)
Plastics	17 (2.9)	57 (3.3)	0.89 (0.52–1.54)	1.03 (0.58–1.83)
Solvents	28 (4.9)	127 (7.3)	0.64 (0.42–0.98)^a^	0.69 (0.45–1.07)
Heavy metals	29 (5.0)	99 (5.7)	0.87 (0.57–1.33)	0.91 (0.59–1.42
Other	47 (8.1)	146 (8.4)	0.96 (0.68–1.36)	0.98 (0.68–1.42)
Self-reported paternal occupational exposure				
Pesticides	51 (8.8)	112 (6.5)	1.40 (0.99–1.98)	1.38 (0.96–1.97)
Plastics	38 (6.6)	131 (7.6)	0.86 (0.58–1.25)	0.90 (0.61–1.03)
Solvents	104 (18)	307 (17.7)	1.02 (0.80–1.31)	1.05 (0.81–1.35)
Heavy metals	114 (19.8)	372 (21.5)	0.90 (0.71–1.14)	0.88 (0.70–1.12)
Other	53 (9.2)	124 (7.2)	1.31 (0.94–1.84)	1.01 (1.00–1.03)

Maternal occupational exposure was adjusted for family history of congenital anomalies, maternal age, maternal education, parity, past abortion, maternal body mass index, gestational or pregestational diabetes, gestational or pregestational hypertension, maternal smoking, and periconceptional folic acid intake

Paternal occupational exposure was adjusted for family history of congenital anomalies, paternal age, paternal education, and paternal smoking

*CHDs* congenital heart diseases, *CI* confidence interval, *OR* odds ratio, *aOR* adjusted odds ratio

^a^*p* **< **0.050

In the multivariate conditional regression analyses, we found no significant association of CHDs with either JEM-assessed or self-reported maternal occupational exposures (Tables 2 and 3). As for fathers, only JEM-assessed probable paternal exposure to polychlorinated organic substances (aOR 2.25; 95% CI 1.05–4.82), phthalates (aOR 1.72; 95% CI 1.11–2.65), biphenolic compounds (aOR 11.5; 95% CI 1.17–113), and solvents (aOR 2.16; 95% CI 1.24–3.76) were significantly associated with the occurrence of CHDs (Tables 2 and 3).

When examining the CHD subtypes separately, we found no significant relationship between either JEM-assessed or self-reported maternal occupational exposure to endocrine disrupting chemicals and CHD subtypes in the offspring (Tables 4 and 5). As for fathers, ventricular septal defects in the offspring were significantly associated with self-reported exposure to pesticides (aOR 1.85; 95% CI 1.10–3.10), atrial septal defects were significantly associated with JEM-assessed phthalate exposure (aOR 2.03; 95% CI 1.14–3.59), and patent ductus arteriosus was significantly associated with self-reported exposure to other types of chemicals (aOR 2.08; 95% CI 1.03–4.18). Finally, paternal solvent exposure was significantly associated to atrial septal defects (aOR 1.82; 95% CI 1.11–3.02) and right ventricle outflow tract obstructions (aOR 3.19; 95% CI 1.01–10.1) (Tables 4 and 5).

**Table 4:** Associations between periconceptional maternal and paternal JEM-assessed occupational exposure and separate CHD subtypes in the offspring

	VSD (*n* = 233)	ASD (*n* = 290)	RVOTO (*n* = 66)	LVOTO (*n* = 37)	PDA (*n* = 108)
	aOR (95% CI)	aOR (95% CI)	aOR (95% CI)	aOR (95% CI)	aOR (95% CI)
JEM-assessed maternal occupational exposure					
Pesticides	1.20 (0.36–4.00)	0.55 (0.07–4.48)	0.74 (0.06–8.97)	–	3.17 (0.46–22.0)
POCs	2.35 (0.28–19.9)	2.14 (0.27–17.2)	–	–	–
Phthalates	0.67 (0.30–1.47)	1.14 (0.63–2.05)	0.97 (0.21–4.48)	4.66 (0.56–38.7)	0.70 (0.18–2.70)
APCs	1.15 (0.40–3.31)	1.50 (0.40–5.65)	0.66 (0.06–7.38)	–	3.17 (0.46–22.0)
BPCs	–	1.55 (0.12–20.6)	–	–	–
Heavy metals	1.03 (0.25–4.27)	1.52 (0.52–4.47)	–	–	–
Solvents	1.26 (0.87–1.83)	1.30 (0.94–1.81)	0.98 (0.41–2.35)	0.62 (0.18–2.23)	1.62 (0.94–2.80)
JEM-assessed paternal occupational exposure					
Pesticides	1.50 (0.70–3.20)	0.73 (0.33–1.60)	0.47 (0.10–2.33)	0.91 (0.08–11.0)	1.01 (0.33–3.13)
POCs	1.18 (0.47–2.96)	1.85 (0.88–3.86)	3.27 (0.69–15.5)	–	1.33 (0.35–5.15)
Phthalates	1.66 (0.84–3.28)	2.03 (1.14–3.59)^a^	2.76 (0.87–8.79)	–	1.85 (0.74–4.59)
APCs	1.59 (0.87–2.91)	1.02 (0.57–1.81)	1.0 (0.33–3.07)	1.65 (0.19–13.8)	1.29 (0.55–3.05)
BPCs	2.92 (0.18–47.7)	–	–	–	–
Heavy metals	1.29 (0.74–2.27)	0.77 (0.46–1.31)	0.55 (0.14–2.09)	0.53 (0.15–1.93)	1.37 (0.60–3.14)
Solvents	1.30 (0.71–2.39)	1.82 (1.11–3.01)^a^	3.19 (1.01–10.1)^a^	1.66 (0.08–36.2)	2.03 (0.91–4.51)

Maternal occupational exposure was adjusted for family history of congenital anomalies, maternal age, maternal education, parity, past abortion, maternal body mass index, gestational or pregestational diabetes, gestational or pregestational hypertension, maternal smoking, and periconceptional folic acid intake

Paternal occupational exposure was adjusted for family history of congenital anomalies, paternal age, paternal education, and paternal smoking

*APCs* alkylphenolic compounds, *ASD* atrial septal defects, *BPCs* biphenolic compounds, *CHDs* congenital heart diseases, *CI* confidence interval, *JEM* job-exposure matrix, *LVOTO* left ventricle outflow tract obstructions, OR odds ratio, *aOR* adjusted odds ratio, *PDA* patent ductus arteriosus, *POCs* polychlorinated organic compounds, *RVOTO* right ventricle outflow tract obstructions, *VSD* ventricle septal defects

^a^*p* **< **0.050

**Table 5:** Associations between maternal and paternal self-reported occupational exposure and CHD subtypes in the offspring

	VSD (*n* = 233)	ASD (*n* = 290)	RVOTO (*n* = 66)	LVOTO (n = 37)	PDA (*n* = 108)
	aOR (95% CI)	aOR (95% CI)	aOR (95% CI)	aOR (95% CI)	aOR (95% CI)
Self-reported maternal occupational exposure					
Pesticides	0.52 (0.11–2.50)	0.93 (0.25–3.51)	–	–	–
Solvents	0.96 (0.50–1.83)	0.71 (0.39–1.30)	0.41 (0.07–2.31)	0.83 (0.09–7.81)	0.44 (0.12–1.65)
Plastics	0.66 (0.26–1.68)	1.30 (0.59–2.89)	5.62 (0.58–54.0)	–	1.37 (0.30–6.33)
Heavy metals	0.99 (0.52–1.92)	0.85 (0.45–1.59)	0.83 (0.14–4.82)	1.26 (0.09–17.8)	0.73 (0.23–2.30)
Other	1.32 (0.75–2.33)	0.91 (0.56–1.50)	1.63 (0.53–5.01)	0.87 (0.13–5.59)	0.99 (0.43–2.25)
Self-reported paternal occupational exposure					
Pesticides	1.85 (1.10–3.10)^a^	1.18 (0.68–2.04)	1.23 (0.37–4.08)	0.27 (0.03–2.50)	1.54 (0.66–3.60)
Solvents	0.80 (0.53–1.23)	1.26 (0.89–1.79)	1.96 (0.41–2.25)	1.43 (0.49–4.20)	1.28 (0.72–2.27)
Plastics	0.58 (0.29–1.14)	1.37 (0.81–2.30)	1.27 (0.39–4.07)	1.32 (0.28–6.12)	0.70 (0.29–1.71)
Heavy metals	0.83 (0.55–1.23)	0.86 (0.60–1.22)	0.88 (0.39–2.00)	0.70 (0.22–2.10)	0.77 (0.44–1.36)
Other	1.08 (0.59–1.98)	1.58 (0.99–2.50)	2.35 (0.84–6.62)	2.02 (0.45–9.09)	2.08 (1.03–4.18)^a^

Maternal occupational exposure was adjusted for family history of congenital anomalies, maternal age, maternal education, parity, past abortion, maternal body mass index, gestational or pregestational diabetes, gestational or pregestational hypertension, maternal smoking, and periconceptional folic acid intake

Paternal occupational exposure was adjusted for family history of congenital anomalies, paternal age, paternal education, and paternal smoking

*ASD* atrial septal defects, *CHDs* congenital heart diseases, *CI* confidence interval, *LVOTO* left ventricle outflow tract obstructions, *OR* odds ratio, *aOR* adjusted odds ratio, *PDA* patent ductus arteriosus, *POCs* polychlorinated organic compounds, *RVOTO* right ventricle outflow tract obstructions, *VSD* ventricle septal defects

^a^*p* **< **0.050

Since Kappa values for JEM-assessed phthalate and polychlorinated organic compound exposure suggested substantial agreement (κ = 0.51), we decided to conduct a post hoc analysis adjusting paternal phthalate exposure for paternal polychlorinated organic compound exposure and vice versa. In this case, odds ratios for paternal exposure to phthalates changed to 1.67 (95% CI 1.01–2.75) and paternal exposure to polychlorinated organic compounds was no longer significant (aOR 1.09; 95% CI 0.58–2.05). Similarly, agreement between paternal solvent and paternal phthalate exposures was fair (*κ* = 0.37). Correcting for the effect of each other, odds ratios changed to 1.80 (95% CI 0.99–3.30) and 1.45 (95% 0.90–2.32) for paternal solvent and paternal phthalate exposure, respectively.

Next, we examined the job titles affected by the aforementioned significant exposures among case fathers, as occupation of mothers was not linked to CHDs in our study. According to our JEM, phthalate exposure was most frequent among case fathers employed as painters, electricians, and hairdressers, biphenolic exposure occurred predominantly among dentists and dental technicians, while pesticide exposure occurred most often among farm workers. Regarding solvents, the most frequently reported occupations were painters and lacquerers, while polychlorinated organic exposure occurred predominantly among electricians.

Finally, the interrater reliability for JEM-assessed and self-reported occupational exposure to pesticides (*κ* value: − 0.011), heavy metals (*κ* value: − 0.024), and solvents (*κ* value: 0.00) suggests poor agreement between methods for mothers and slight agreement for paternal pesticide (*κ* value: 0.2), paternal heavy metal (*κ* value: 0.14), and paternal solvent (*κ* value: 0.14) exposures.

## Discussion

In our case–control study, we examined the effect of occupational exposures to endocrine disrupting chemicals on the occurrence of CHDs. Controls were matched to cases in respect of their date of birth and gender of newborns, and settlement size of family’s residence. Maternal exposure to endocrine disrupting chemicals did not result in a significant increase of CHD occurrence in the offspring. Regarding paternal exposure, we found a positive relationship between JEM-assessed paternal polychlorinated organic compound, phthalate, biphenolic compound, and solvent exposures and the entire spectrum of CHDs. Subtypes, such as ventricle septum defects, were positively associated with self-reported paternal pesticide exposures, while atrial septal defects were positively associated with JEM-assessed paternal exposure to phthalates and solvents. Furthermore, solvents were also associated to a more frequent occurrence of right ventricle outflow tract obstructions.

Our results are in concordance with other studies regarding paternal phthalate (Snijder et al. 2012; Nicoll 2018), biphenolic compound (Snijder et al. 2012), solvent (Gilboa et al. 2012), polychlorinated organic compound (Nicoll 2018), and pesticide exposures (Wilson et al. 1998). Snijder et al. (2012) found similar association between CHDs in the offspring and paternal phthalate exposure, also using van Tongeren et al.’s (2002) JEM for occupational risk assessment (Snijder et al. 2012). This relationship is further corroborated by another study with similar results (Nicoll 2018). In regards of biphenolic compounds, Snijder et al. (2012) also found a significant association with CHDs and relatively wide confidence intervals, which may be explained by the overall low exposure levels in both studies. Biphenolic compounds have not been widely associated with congenital heart defects in humans, however, animal studies suggest that these compounds may interfere with the normal development of the heart (Lombó et al. 2015). The fact that patent ductus arteriosus was associated with self-reported paternal exposure to other non-specified substances suggests the possible presence of other occupational hazards that were unaccounted by the current study. As of maternal exposure, we were not able to identify any occupational risk factors, which is in line with the results of Snijder et al. (2012). Conversely, other studies found a significant relationship for maternal exposure to certain solvents, polyaromatic hydrocarbons, pesticides, alkylphenolic compounds, and heavy metals (Baldacci et al. 2018; Nicoll 2018), which indicates that our results concerning maternal occupational exposure should be interpreted with caution. When comparing exposure prevalence of mothers and fathers, we found higher paternal exposure prevalence to chemicals compared to mothers, which may explain why the effect of maternal occupational exposure was less prominent in our study.

In regards of occupations that may increase the occurrence of CHDs in the offspring, previous studies found similar job titles as the ones identified by us (Snijder et al. 2012). Phthalates are usually found in plastics, cosmetics, construction materials, printing inks, and other products used by electricians, painters, hairdressers and those working in the printing industry. Polychlorinated organic compounds are utilized regularly in electrical equipment, pigments, and plasticizers (EPA 2019). Even though biphenolic compounds may occur in plastics for dental sealants used by dentists and dental technicians (EPA 2019), our results should be interpreted cautiously because of small numbers and crude risk assessment. Farm workers are frequently exposed to pesticides during the preparation and application of these products, or during the cleaning-up of spraying equipment (IARC 2012; Bergman et al. 2013; Pecht et al. 2018), which might offer a possible explanation for our results. Painters and lacquerers are exposed to a wide variety of solvents during their work, namely toluene, aliphatic and alicyclic hydrocarbons solvents, and aromatic hydrocarbons solvents (Hadkhale et al. 2017). Our results should be interpreted with caution as our JEM was not validated by actual measurement data, and JEM-assessed exposure to phthalates for instance is often not corroborated by biological monitoring data among people working as hairdressers and cosmetologists, indicating that individuals involved in these occupations are not necessarily exposed to higher levels of phthalates than the background exposure level (Bonde 2020). It must be noted, however, that JEM-assessed exposure to pesticides, solvents, and metals, are often more valid based on the results of biological monitoring (Bonde 2020).

JEMs and self-reported exposure assessment show great variance ranging from no to substantial agreement, when compared to the results of expert assessment (Teschke et al. 2002). In our study, the consistency of JEM-assessed and self-reported occupational exposure to pesticides, heavy metals, and solvents exhibited poor agreement for mothers, and slight agreement for fathers. Other studies found moderate-to-substantial agreement between these two methods with lower agreement in regards of pesticides among those aged over 50 (Adegoke et al. 2004). The agreement of methods is influenced by several factors. JEMs are affected by misclassification, and they do not take into consideration variance in exposure within a job title. Moreover, neither JEM-assessed nor self-reported occupational exposure assessment account for exposures outside of work (e.g. household) (Teschke et al. 2002). Since background exposure affects the whole population and not just individuals employed in certain occupations, its confounding effect was probably not as significant in our study. Accuracy of self-reported data are also affected by other factors that do not influence JEMs. On one hand, participants are more likely to report exposures that are easy to sense. On the other hand, workers may not be familiar with the names of specific chemical compounds (Teschke et al. 2002), and may not report certain occupational risk factors that they were exposed to otherwise. In our study, self-reported exposures were listed in ways that were simple to recognize, which unfortunately may have led to the overestimation of true exposure. According to our study population, JEM-assessed exposure was lower compared to self-assessed exposure. This gap between assessment methods may indicate that overestimation was indeed present for self-reporting. Nevertheless, higher self-reported occupational exposure assessment was generally not linked to more frequent occurrence of CHDs, except for ventricle septal defects. This, however, was not corroborated by the results of the JEM-assessment. Self-reported occupational exposure assessment is rarely used for the analysis of CHDs in the offspring. Although a study relying on self-reported maternal exposure assessment to pesticides found that pesticides increase the occurrence of transposition of the great arteries among their offspring, this could not be verified by a later study relying on expert assessment (Rocheleau et al. 2015). Taking all this into consideration, the results of studies examining CHDs in the offspring may be sensitive to the type of occupational assessment method used. Selection of occupational risk assessment method should depend on the job, as people involved in certain occupations, for instance farmers, who purchase pesticides regularly, may have a better understanding of the chemicals compared to gardeners, who may work with products purchased by someone else (Teschke et al. 2002).

A potential strength of our study is that controls were adjusted not just for gender and date of birth but for settlement size as well, further decreasing the effect of possible confounders. However, since the etiology of CHDs is not fully understood yet, we cannot rule out the effect of residual confounding. Even though case–control studies are not suited to measure absolute risk directly, odds may be comparable to absolute risk in case of rare diseases and when controls are recruited shortly after the selection of cases to prevent bias deriving from dynamic populations (Vandenbroucke and Pearce 2012). Since controls were interviewed shortly after the inclusion of cases, the effect of the latter may have been kept at a minimum in our study. As prospective studies for congenital malformations require large sample sizes because of the low prevalence of these diseases, case–control studies are still considered as one of the most reliable methods to estimate causal relationship of CHDs’ risk factors.

Finally, we must address several limitations arising from the study design itself. First of all, considering the rarity of CHDs in the offspring, more data would have been helpful in our population-based case–control study. In our conditional regression analyses, several consecutive comparisons were made, which may have increased the potential risk of false-positive results. Examining the entire spectrum of CHDs is also somewhat problematic as the individual malformations may have different etiologies and a different relationship with exposure to endocrine disrupting chemicals. The subgroup analysis also narrowed down the sample size, further enhancing the uncertainty of our results. Furthermore, heavy metals and pesticides were not examined individually, so the effect of certain non-hazardous chemicals may have mitigated the effect of other potentially dangerous chemicals. Moreover, in case of certain occupations, such as electricians and painters, it is impossible to disentangle the effect of individual endocrine disrupting chemicals, for instance solvents, polychlorinated organic compounds and phthalates, as those employed in these occupations are exposed to multiple compounds at the same time. Case–control studies are also susceptible to selection and recall bias. Selection bias may have influenced the precision of our results, since only liveborn cases were included in our study, resulting in the exclusion of certain more severe cases of CHDs. The effect of recall bias, however, may be minimal in case of JEM assessment as cases and control parents were only asked about their periconceptional occupation shortly after the reporting of cases which is customarily done within one month of diagnosis. In case of self-reported exposure, however, the role of recall bias cannot be ruled out. Finally, it must be noted that our results were not verified by more reliable exposure assessment data, such as environmental and biological monitoring. Thus, our results should be interpreted with caution due to the limitations of JEMs and self-reported occupational exposure assessment and should be considered reassuring, as significant results were overall scarce.

Based on our results, maternal occupational exposure did not exert a significantly adverse effect in regards of CHDs in the offspring. As for fathers, certain exposure, such as phthalates, biphenolic compounds, solvents, and pesticides may increase the occurrence of the entire spectrum of CHDs and individual CHD phenotypes in the offspring. The identification of occupations that increase the risk of certain diseases, such as CHDs, may give policy makers and stakeholders incentive to make changes. For instance, implementing and strengthening preventive measures (e.g. airway protection against phthalate containing particles (Szewczyńska et al. 2019)) at the workplace may decrease exposure of workers. The improvement of referral efficacy may increase the cost-effectiveness of screening tests (Pinto et al. 2014) and decrease the proportion of unrecognized cases. As of now, CHDs are not always recognized by prenatal screening tests. Moreover, newborns affected by CHDs may appear healthy at birth and be discharged from the hospital (CDC 2018), resulting in delayed treatment. Paying closer attention to occupational history may help to identify potential risk groups for closer observation, resulting in better recognition and immediate treatment of patients if necessary.

## Conclusion

In our gender, age, and settlement size matched case–control study, the entire spectrum of CHDs was significantly associated with JEM-assessed paternal phthalate, biphenolic compound, polychlorinated organic compound, and solvent exposures after adjustment for confounders. Regarding CHD subtypes and paternal occupational exposure, self-reported pesticide exposure was linked to ventricular septal defects, while JEM-assessed exposure to phthalates remained significant for atrial septal defects. Paternal solvent exposure was linked to the higher occurrence of not only atrial septal defects but right outflow tract obstructions in the offspring as well. On the other hand, maternal occupational exposure was not significantly associated with CHDs in the offspring in our study. When comparing the consistency of JEM-assessed and self-reported exposure to pesticides, heavy metals, and solvents directly, we found poor agreement between methods in case of mothers and slight agreement in case of fathers. In overall, our results should be considered reassuring, as parental occupational exposure to endocrine disrupting chemicals seems to have a minor impact on the occurrence of CHDs in the offspring. To better assess the role of occupational exposure, the results of biological and environmental monitoring should also be taken into consideration to more precisely explore exposure levels at an individual level.

## Data Availability

The data that have been used are confidential.
